# Metabolic reprogramming in hepatic stellate cells: unveiling a novel therapeutic target for liver fibrosis

**DOI:** 10.3389/fphys.2026.1803238

**Published:** 2026-07-15

**Authors:** Bingbing He, Ning Wang, Ruijuan Yan, Junzhe Jiao, Haibo Zhang, Qian Huang, Qi Xi, Zhanjie Chang, Xi Guan, Shuguang Yan, Jingtao Li

**Affiliations:** 1 Shaanxi University of Chinese Medicine, Xianyang, China; 2 Departments of Infectious Disease, The Affiliated Hospital of Shaanxi University of Chinese Medicine, Xianyang, China; 3 College of Basic Medicine, Shaanxi University of Chinese Medicine, Xianyang, China; 4 Department of Pathology, Shaanxi University of Chinese Medicine, Xianyang, China; 5 Department of Warm Disease, Shaanxi University of Chinese Medicine, Xianyang, China; 6 Key Laboratory of Gastrointestinal Diseases and Prescriptions in Shaanxi Province, Shaanxi University of Chinese Medicine, Xianyang, China

**Keywords:** energy metabolism, hepatic stellate cells, liver fibrosis, matrix stiffness, mechanotransduction

## Abstract

Liver fibrosis represents a pathological reparative process for the liver that occurs due to persistent harm, closely linked to sustained liver damage and inflammation. In healthy livers, hepatic stellate cells remain quiescent; however, following liver injury, their transformation into myofibroblasts results in extracellular matrix deposition, thereby worsening the developing stages of hepatic fibrosis. According to research, the main cause of liver fibrosis is the activation of hepatic stellate cells. Significant progress has been made in the study of the development and regression mechanisms of liver fibrosis in recent years, as well as a better understanding of the function of hepatic stellate cells in the fibrous process. The metabolic reprogramming of hepatic stellate cells is a crucial factor facilitating their activation and is essential for their effector roles and anabolic requirements; current research indicates that mechanosignaling is a significant regulator of cellular metabolism. This study summarizes the impact of hepatic stellate cell energy metabolism on fibrosis progression, examines the relationship between mechanomechanics and cellular metabolism, and discusses the therapeutic possibility of targeting cellular energy metabolism.

## Graphical Abstract

**Figure:**
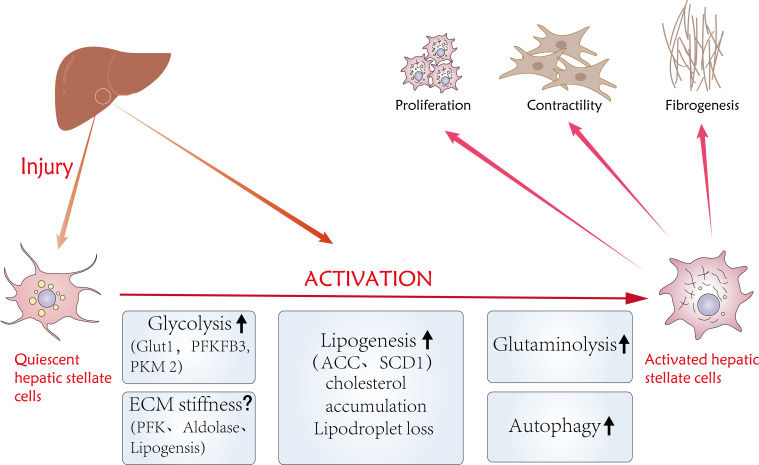
Targeting intracellular metabolic pathways in hepatic stellate cells: impact on liver fibrosis. Vitamin A-rich hepatic stellate cells in a resting state under normal conditions, After liver damage has occurred, Hepatic stellate cells begin to activate, triggering their transdifferentiation into myofibroblasts, During their activation, matrix stiffness, glycolysis, glutamine metabolism, *de novo* lipogenesis, cholesterol accumulation, loss of lipid droplets, and autophagy all contribute to the promotion of the activation process, Proliferation, contractility, and the occurrence of fibrosis are characteristics of myofibroblasts.

## 1 Introduction

In their quiescent state, hepatic stellate cells (HSC), which are mesenchymal cells located in the perisinusoidal region of the hepatic sinusoids, are responsible for storing vitamin A and maintaining the homeostasis of the hepatic extracellular matrix (ECM) (Tsuchida and Friedman, 2017). Upon hepatic injury, they activate into myofibroblasts, characterized by excessive collagen synthesis and impaired degradation(ultimately driving the progression of liver fibrosis) (Higashi et al., 2017). Cellular metabolism is the fundamental process that sustains living activities. It primarily encompasses glucose, lipid, and glutamine metabolism, which are interrelated and collaborate to uphold cellular homeostasis. An essential component of HSC activation is metabolic reprogramming. Damage signals cause HSC to transition from an inactive state, which relies on the fatty acid oxidation, to a metabolic state primarily regulated by glycolysis and glutamine metabolism. This shift meets the energy and compositional requirements for activation (Trivedi et al., 2021). Furthermore, mechanical forces such as fluid shear, matrix stiffness, and cellular tensile forces can activate intracellular signaling pathways through cell surface mechanosensors, thereby regulating cell proliferation, migration, differentiation, and apoptosis. This regulation relies on energy generated by the metabolic conversion of nutrients.

Liver fibrosis is a pathological condition characterized by abnormal ECM accumulation and extensive tissue overgrowth, leading to structural and functional abnormalities of the liver. Liver fibrosis has a complex etiology that includes common causes such as non-alcoholic fatty liver disease/non-alcoholic steatohepatitis, high levels of alcohol consumption, and chronic hepatitis B or C virus infections, as well as uncommon ailments, including primary/secondary cholangitis, hemochromatosis, Wilson’s disease, and autoimmune hepatitis (Xu et al., 2023). While liver fibrosis can be managed and potentially reversed by addressing the underlying cause, uncontrolled progression leads to the decompensated stage of cirrhosis, resulting in life-threatening complications such as hepatic encephalopathy, portal hypertension, and gastrointestinal bleeding (Tsuchida and Friedman, 2017). Statistics indicate that liver cirrhosis results in 1 million fatalities globally each year (Higashi et al., 2017). Currently, the treatment of liver fibrosis is no longer limited to therapies targeting the underlying cause. Resmetirom and semaglutide have received conditional approval from the US Food and Drug Administration (FDA) for use in adult patients with noncirrhotic MASH accompanied by moderate to severe fibrosis (stages F2–F3) (Harrison et al., 2024; Sanyal et al., 2025).

Recent studies indicate that HSC activation necessitates substantial energy support, promoting metabolic reprogramming in HSCs to meet their energy and biosynthetic precursor requirements during activation (Bai He Ti Ya Er and Guo, 2021). Therefore, regulating metabolic reprogramming in HSCs represents a potential therapeutic avenue for the suppressive treatment of liver fibrosis. This review aims to delineate the intricate relationship between HSC substance metabolism and its activation. It focuses on three key aspects. First, it explores how glycolysis, lipid metabolism, and glutamine metabolism play distinct roles in driving the fibrotic activation of HSCs. Second, it examines the emerging role of matrix stiffness and mechanotransduction in regulating cellular metabolism. Third, it considers the therapeutic potential of targeting HSC substance metabolism in anti-fibrosis strategies (Gilgenkrantz et al., 2021). Current evidence suggests that directly targeting interconnected metabolic pathways through a multi-target combinatorial strategy is the most effective therapeutic approach for combating liver fibrosis in future studies.

### 1.1 HSC activation drives liver fibrosis

HSCs are a type of nonparenchymal liver cell located in the subendothelial region of the Disse space that regulates metabolic reprogramming. When the liver is healthy, they are characterized as nonproliferative, quiescent cells resembling adipocytes, containing numerous lipid droplets and abundant retinyl esters within their cytoplasmic lipid droplets, thereby serving as the principal reservoir of vitamin A in the body.

When liver damage occurs, hepatocytes, Kupffer cells, and hepatic sinusoidal endothelial cells secrete substantial quantities of cytokines that facilitate the transcription of profibrotic genes HSC nuclei via the downstream signaling pathways. This process results in the phenotypic transformation of HSC from adipocytes to myofibroblasts, endowing them with functions such as proliferation, contraction, and angiogenesis. Therefore, activated HSCs, referred to as myofibroblasts, synthesize ECM constituents including α-smooth muscle actin (α-SMA) and collagen types I and III. They also enhance the expression of tissue inhibitors of metalloproteinases (TIMPs) and suppress matrix metalloproteinase (MMP) activity. They have the capacity to intensify the pro-inflammatory response, react to pro-inflammatory stimuli, and modify the immune milieu, resulting in ECM accumulation and the progression of fibrosis (Tsuchida and Friedman, 2017). As liver injury resolves, myofibroblasts originating from HSCs decrease via apoptosis or revert to a “quiescent” state; nevertheless, these previously active “quiescent” states may possess distinct epigenetic characteristics that render them more prone to reactivation following further liver injury (Trivedi et al., 2021). Thus, HSC activation is a major driver of liver fibrosis. HSC transformation into myofibroblasts requires a substantial energy supply, prompting HSCs to undergo intracellular metabolic reprogramming to satisfy the material and energy requirements of activation.

**Figure 1:**
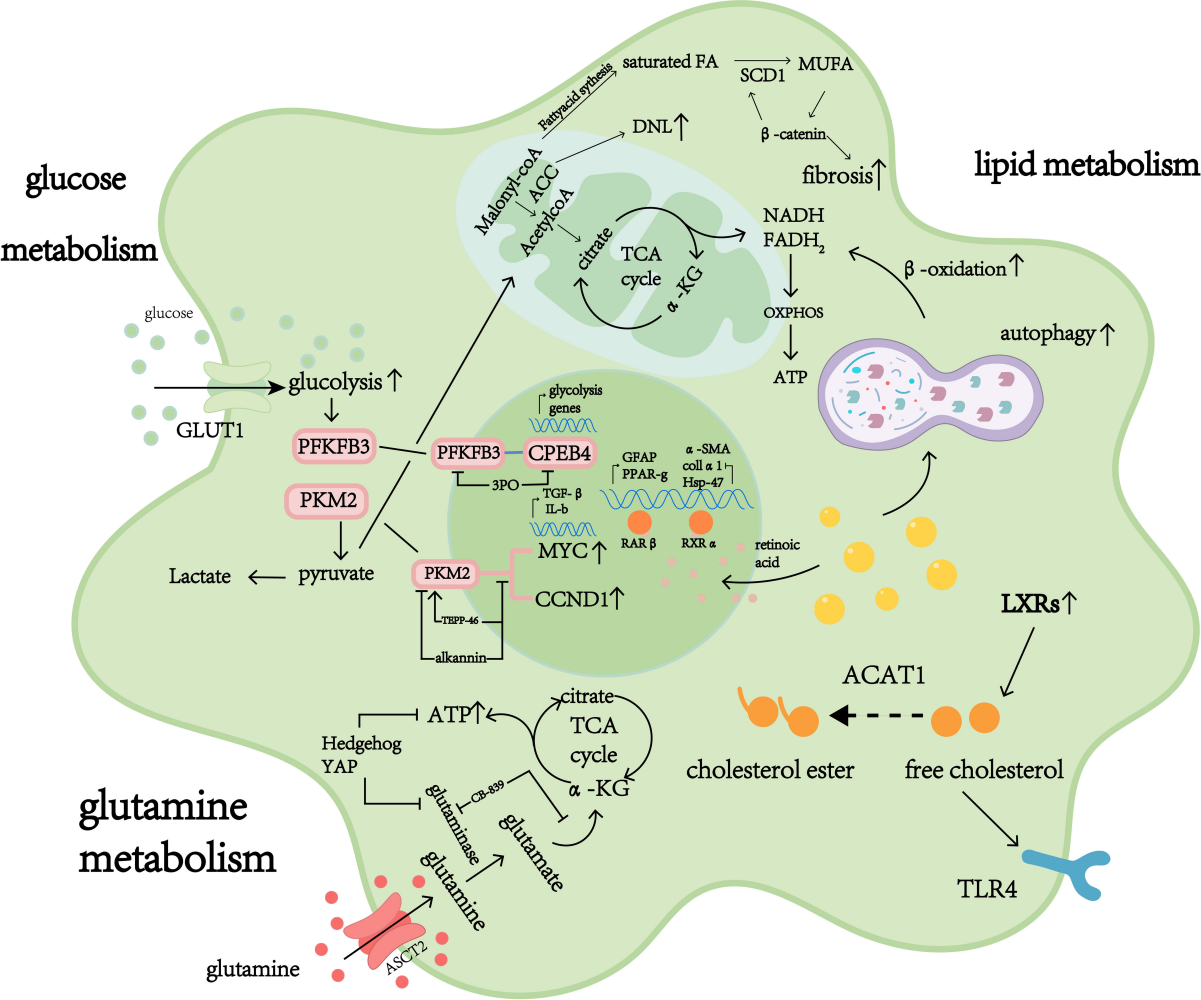
Metabolic reprogramming in fibrogenic hepatic stellate cell activation. The activation of HSCs causes significant phenotypic changes, and activated HSCs increase glycolysis, the tricarboxylic acid cycle (TCA), and catabolism of lipid droplets to fuel their phenotypic transformations through an increase in glucose transporter 1 (GLUT1). Pyruvate Kinase M2(PKM2) enters the nucleus to promote the HSCs’ promoter MYC Proto-Oncogene(MYC), Cyclin D1(CCND1), and Fructose-2,6-Bisphosphatase 3(PFKFB3), which supports the expression of glycolytic genes by binding to Cytoplasmic Polyadenylation Element Binding Protein 4(CPEB4). Decomposed lipid droplets can be used for β-oxidation to provide energy. Glutamine metabolism is another source, where glutamate and α-ketoglutarate, which are produced by the metabolism of glutamine, enter the TCA cycle to generate energy. In addition, HSCs can upregulate the autophagy pathway to derive energy from lipid droplets storing retinol. Activated HSCs are enriched in free cholesterol, which promotes TLR4 expression and makes HSCs more sensitive to transforming growth factor-β (TGF-β) signaling. ACAT1, acetyl coenzyme A acetyltransferase; FXR, farnesol receptor; LXR, liver X receptor; TLR4, toll-like receptor 4.

Sugars, lipids, and proteins, the three fundamental nutrients vital to the human body, serve an indispensable and pivotal function in biological processes. Their metabolism can provide ATP and small molecules (e.g., amino acids) for cellular proliferation, differentiation, transformation, apoptosis, and autophagy (Trivedi et al., 2021). Recent investigations indicate that the metabolic remodeling of glycolysis, lipid metabolism, and glutamine metabolism is necessary for HSC activation (Du et al., 2018; Mejias et al., 2020; Trivedi et al., 2021) (**Figure 1**).

## 2 The activation of HSC is contingent upon the reprogramming of metabolic substances

### 2.1 Glycolysis and HSC activation

Glucose metabolism is a crucial catalyst for HSC activation, primarily via enhancing glucose metabolism to fulfill the material and energy demands for activation. Glycolysis is the process of cleaving a glucose molecule into two pyruvate molecules, with pyruvate as the final product. Pyruvate enters the mitochondria in the presence of oxygen, where it is oxidation to acetyl-coenzyme A (acetyl-CoA) and participates in the TCA. In the absence of oxygen, pyruvate is converted to lactate, and anaerobic oxidation becomes the sole pathway for ATP synthesis in environments devoid of oxygen or in cells lacking mitochondria, such as erythrocytes and thyroid glands (Chandel, 2021). Despite glycolysis yielding far less ATP than oxidative phosphorylation, it generates ATP at a considerably higher pace, making glycolysis more adept at meeting the fast energy demands necessary for phenotypic flipping in HSCs. Consequently, during the conversion of HSCs into myofibroblasts, even when ample oxygen is present, the cells prefer anaerobic glycolysis over oxidative phosphorylation to generate ATP. This adaptation facilitates energy supply for the phenotypic transition by upregulation glycolysis, a phenomenon known as the Warburg-like effect.

Glycolysis involves three principal enzymes and one transport protein: hexokinase2(HK2), phosphofructokinase-1(PFK1), PKM2, and glucose transporters (GLUTs). The transfer of glucose is the rate-limiting factor in ATP synthesis during HSC glucose metabolism, mostly facilitated by GLUT. Elevated mitochondrial activity and glyceraldehyde-3-phosphate dehydrogenase (GAPDH) expression, together with a substantial rise in glucose absorption, were seen in both human activated HSCs and cultured activated rat HSCs (Gajendiran et al., 2018). Hepatic GLUT1 expression was shown to be markedly elevated in methionine-choline-deficient (MCD) diet, bile duct ligation (BDL)-modeled mice with chronic liver damage as well as liver fibrosis, and CCL_4_-modeled animals with acute liver injury (Chen et al., 2012). In fibroblast cell lines and primary cells, TGF-β induces GLUT1 expression, and inhibition of GLUT1 function or expression diminishes TGF-β-mediated fibrogenesis (Andrianifahanana et al., 2016). The results, as mentioned above, indicate that GLUT targeting might be a useful treatment approach for liver fibrosis. A substantial quantity of genes exhibiting differentially expressed in resting HSCs versus myofibroblasts are linked to metabolic processes. In cultured HSCs, a notable elevation in the manifestation of glycolytic enzymes (HK2, PFKFB3, PKM2) and proteins that transport glucose and lactate was observed in myofibroblasts relative to the resting phenotype. The synthesis of fructose-2,6-bisphosphate (F2,6BP) is catalyzed by PFKFB3, the strongest activator of the enzyme PFK1, which limits the rate of glycolysis, a primary initiator of glycolysis.

Research indicates that PFKFB3 and glycolysis, crucial for HSC activation, are markedly increased in the fibrotic liver tissues of both humans and rodents (Mejias et al., 2020). PFKFB3 interacts with the RNA-binding protein CPEB4, leading to increased glycolytic activity in HSCs in fibrotic livers compared to normal livers. Glycolysis-driven metabolic reprogramming in HSCs promotes myofibroblasts’ phenotypic transformation, enhances ECM production and storage, thereby worsening hepatic fibrosis, whereas inhibition of PFKFB3 mitigates these alterations. Mejias et al. have validated the antifibrotic effects of the CPEB4 inhibitor 3PO in various HSC models. In mouse HSCs, 3PO treatment effectively inhibited HSC activation. In human primary HSCs, 3PO treatment resulted in a 51% reduction in F2,6BP levels and significantly downregulated the expression of pro-fibrotic proteins such as α-SMA, Platelet-Derived Growth Factor Receptor Beta(PDGFR-β), and Vascular Endothelial Growth Factor (VEGF). In a mouse model of bile duct ligation, administration of 3PO immediately after surgery prevents HSC activation and reduces liver fibrosis. In addition, PFKFB3 is highly expressed in the liver tissue of patients with cirrhosis (Mejias et al., 2020).

PKM2 serves as a vital regulatory locus in glycolysis (Yang et al., 2012). The expression of PKM2 is markedly elevated in cancerous cells and can stimulate glycolysis. PKM2 appears in mammals primarily in two forms: dimerization and tetramerization. Because of its strong PK activity, the PKM2 tetramer catalyzes the transformation of phosphoenolpyruvate into pyruvate, as opposed to the PKM2 dimer, which may translocate to the nucleus, has protein kinase activity, and has minimal PK activity, where it participates in protein modification and regulates the transcription of downstream genes. Research has shown that PKM2 primarily exists as a low-activity dimer in activated HSCs and can translocate to the cell nucleus (Zheng et al., 2020; Luo et al., 2025). PKM2 dimers promote HSC activation by regulating histone H3K9 acetylation, thereby upregulating the expression of transcription factors such as MYC and CCND1 (Zheng et al., 2020). The PKM2 agonist TEPP-46 induces PKM2 tetramerization, retaining PKM2 in the cytoplasm and thereby effectively suppressing the expression of HSC activation markers (Zheng et al., 2020). Importantly, in a CCl_4_-induced mouse model of liver fibrosis, treatment with TEPP-46 significantly reduced hepatic hydroxyproline content, Sirius red-positive area, and the expression of fibrosis markers such as α-SMA and collagen type I alpha 1 chain (COL1A1) (Zheng et al., 2020). The PKM2 inhibitor shikonin also blocks PKM2 dimerization, inhibits HSC activation, and reduces liver fibrosis (Zheng et al., 2020).

The activation and metabolic reprogramming of HSCs exhibit significant regional heterogeneity, which is closely associated with the microenvironment of the specific liver compartment in which they reside (Cogliati et al., 2023).In a healthy liver, the periportal region is enriched with a subtype of 1-HSCs that are rich in vitamin A and primarily involved in extracellular matrix remodeling and glucose metabolism; in contrast, the central venous zone contains a subpopulation of HSCs that are more readily activated (Cogliati et al., 2023). However, as fibrosis progresses, this compartment-dependent metabolic balance is disrupted. The study found that fibrosis-associated genes and HSC markers were significantly enriched in the central venous zone, and that HSCs in the central venous zone initiated glycolysis upon stimulation with Platelet-Derived Growth Factor(PDGF), By enhancing histone H3K9 acetylation, the expression of Ras-Related Protein Rab-31(RAB31), a GTPase involved in vesicular transport, is upregulated, leading to the massive release of extracellular vesicles (EVs) rich in fibrogenic proteins, EVs transmit signals between cells, ultimately amplifying liver fibrosis in a spatially specific manner (Khanal et al., 2024). In summary, the regional heterogeneity of HSC metabolic reprogramming offers a new perspective for the precision treatment of liver fibrosis. Future research should focus on elucidating the molecular regulatory networks underlying metabolic reprogramming of HSCs in different compartments; developing combined therapeutic strategies targeting key enzymes involved in glycolysis (such as PFKFB3 and PKM2) and proteins associated with EV release (such as RAB31) in these regions; such research will contribute to the development of precision anti-fibrotic strategies based on HSC compartmental heterogeneity.

In summary, the metabolic reprogramming of HSCs is the fundamental pathophysiology underlying the development of liver fibrosis. After liver damage caused by stimuli, HSCs become activated and develop into myofibroblasts, acquiring the capacity to proliferate, contract, and produce ECM. This activation frequently necessitates a substantial metabolic paradigm shift: aerobic glycolysis replaces oxidative phosphorylation in glucose metabolism, and this remodeling of metabolism satisfies the significant energy requirements of HSC activation. Research indicates that PKM2 dimers may migrate to the nucleus and function as transcriptional coactivators to regulate gene expression (Yang et al., 2012). Moreover, it has been established that LPS-induced PKM2 dimer translocates to the nucleus, forming a complex with Hypoxia-inducible factor-1α (HIF-1α), which directly interacts with the promoters of pro-inflammatory genes, including Interleukin-1 Beta(IL-1β), thereby augmenting their transcription and facilitating the polarization of M1-type macrophages and the synthesis of pro-inflammatory mediators, such as Tumor necrosis factor-α (TNF-α) and IL-β (Palsson-McDermott et al., 2015). Research indicates that in tumour cells, HIF-1 can directly associate with the Hypoxia Response Element(HRE) in intron 1 of the PKM2 gene, thereby upregulating PKM2 gene transcription. Through the facilitation of HIF-1 binding and p300 recruitment to the promoters of HIF-1 target genes, this interaction with the HIF-1α component increases HIF-1’s transcriptional activity, promoting H3K9 acetylation and the upregulation of glycolytic gene expression. PKM2 amplifies the Warburg effect by participating in a positive feedback loop, thereby augmenting glycolysis and fostering tumor metabolic phenotypes via the overexpression of GLUT1, lactate dehydrogenase (LDH), and pyruvate dehydrogenase kinase1 (PDK1) (Luo et al., 2011; Du et al., 2022). So, is HSC activation also associated with PKM2 dimerization into the nucleus to bind HIF-1α? Can HIF-1α inhibition reverse glycolytic reprogramming? The precise molecular mechanism behind the metabolic reprogramming after HSC activation remains unidentified and necessitates additional investigation.

### 2.2 Lipid metabolism and HSC activation

Lipid metabolism encompasses the processes of intake, synthesis, catabolism, transport, and storage of fats, such as triglycerides and cholesterol, inside the body. Lipid metabolism disorders (e.g., excessive fat intake, heightened synthesis, or diminished catabolism) contribute to various diseases, including metabolic syndrome, obesity, and type 2 diabetes mellitus (Petrenko et al., 2023). Lipid metabolism primarily occurs in the liver; disruotions in this process lead to the abnormal accumulation of fat in the liver, thereby causing hepatocellular damage and chronic liver inflammation.

#### 2.2.1 Autophagy and lipid droplet loss

Quiescent HSCs are identified as adipocytes containing substantial intracellular lipid droplets (LD) and substantial amounts of retinyl esters in their LDs in the cytoplasm, which serve as reservoirs of vitamin A as well as represent the principal source of this vitamin. Consequently, one of the characteristics of HSCs in both healthy and injured livers is their ability to regulate vitamin A homeostasis (Trivedi et al., 2021). HSCs retain 50–95 percent of the body’s vitamin A, comprising retinol as well as its metabolites (Blomhoff and Blomhoff, 2006). Retinyl esters (REs), which make up 30% to 50% of the cytoplasmic LD composition in HSCs, are the form in which more than 95% of vitamin A analogs are stored (Senoo et al., 2010). Research indicates that vitamin A in HSCs correlates with several periplasmic proteins connected to LD surfaces. These include adipocyte differentiation-related protein (ADRP) (Fukushima et al., 2005), adipophilin (Straub et al., 2013), perilipin5 (Lin et al., 2018), adipose triglyceride lipase (ATGL) (Eichmann et al., 2015), and comparative gene identification-58 (CGI-58) (Eichmann et al., 2015), in addition to liver-fatty acid-binding protein (L-FABP) (Chen et al., 2013). Increased expression of periplasmic proteins correlates with diminished activation of HSCs, likely via stabilizing retinol droplets, so decreasing the catabolism of retinol into fatty acids for energy production (Zhou et al., 2020). Retinol preserves the quiescent HSC phenotype chiefly via interactions with the Retinoic Acid Receptor Beta(RARβ) and the Retinoid X Receptor Alpha(RXRα)nuclear receptors (Wang et al., 2004). Treatment of HSCs with vitamin A keeps the markers of quiescent HSCs expressed (Glial Fibrillary Acidic Protein(GFAP) and Peroxisome Proliferator-Activated Receptor Gamma(PPAR-γ)) and prevents their activation, while concurrently suppressing myofibroblast markers (α-SMA, Col1A1, and Heat Shock Protein 47(HSP-47)) (Yoneda et al., 2016). In addition, retinol treatment promotes the return of partially activated HSCs to their quiescent state, where LDs are lost in large numbers during the transformation of HSCs into myofibroblasts (Zhou et al., 2020). LD loss happens histologically in two stages. The first stage is that the LDs are progressively broken down into smaller units. As the cell divides, they are reallocated from the perinuclear area into the newly created cell. And the amount of RE that makes up the LDs is now decreasing, accompanied by an increase in triglyceride levels. The second stage is that the LDs gradually become smaller through disintegration until they completely transdifferentiate into myofibroblasts, and thus disappear. The primary characteristic of HSC activation is the progressive degradation of LD within the cytoplasm to a diminished size until they are eliminated. In contrast, their activation correlates with the depletion of intracellular LDs. Nonetheless, to date, no functional correlation has been shown between LD depletion and cellular activity (Hernández-Gea et al., 2012).

Cellular autophagy plays a crucial role in liver fibrosis. Mice with hepatocytes or endothelial cells deficient in Atg5 or Atg7 exhibit higher levels of liver fibrosis (Ni et al., 2014; Hammoutene et al., 2020). A recent study indicates that cellular autophagy initiates intracellular lipolysis, with the resultant fatty acids utilized for energy production in hepatocytes. During autophagy in hepatocytes, lysosomes degrade intracellular lipids (Singh et al., 2009). Following liver injury generated by CCl_4_ and TAA, activated HSCs promote fibrotic markers and enhance the expression of autophagy-related genes (Hernández-Gea et al., 2012). This outcome indicates that the activation of autophagy is essential for sustaining HSCs undergoing phenotypic transition following metabolic stress during hepatic damage. This perspective aligns with findings from an alternative cellular investigation, wherein the inhibition of autophagy in HSCs curtailed fibrogenesis *in vivo* following the administration of a nonspecific autophagy inhibitor, resulting in a notable decrease in fibrogenic genes (α-SMA, collagen1α1, collagen1α2, PDGFR-β, and MMP-2), without affecting cellular activity (Thoen et al., 2011). The data, as mentioned above, demonstrate that autophagy stimulates HSC activation, enhances ECM formation, and support energy production. Moreover, autophagy is contingent upon ATP (Liu et al., 2025). Inhibition of autophagy reduces the rate of mitochondrial β-oxidation and energy production (Singh et al., 2009; Heaton et al., 2010). Autophagy-deficient HSCs are unable to break down lipid droplets in the cytoplasm by acid lipase, leading to accumulation of lipid droplets and a decrease in free fatty acid content, and reducing mitochondrial β-oxidation and energy production (Hernández-Gea et al., 2012). These investigations indicate that autophagy can supply the requisite energy for HSC activation by catabolizing LDs, producing free fatty acids, and facilitating mitochondrial β-oxidation. Additional suppression of HSC activation by inhibiting autophagy, which diminishes lipid droplet catabolism, free fatty acid synthesis, and mitochondrial β-oxidation, may provide a novel approach in combating liver fibrosis.

#### 2.2.2 *De novo* lipogenesis and HSC activation

In organs like the liver, a process known as *de novo* lipogenesis (DNL) coverts precursors such as amino acids and carbohydrates into fatty acids, which are then further esterified to triglycerides. In Metabolic dysfunction-associated steatotic liver disease (MASLD), DNL’s abnormal enlargement is a pivotal mechanism for excessive hepatic fat accumulation (Liao et al., 2024). Previous studies indicate that diminishing lipid buildup may impede the activation of HSCs and consequently influence liver fibrosis (Wobser et al., 2009). Acetyl-CoA carboxylase (ACC) facilitates DNL in hepatocytes and is essential for regulating its rate. ACC further governs fatty acid β-oxidation (Wang et al., 2022). The transformation of acetyl-CoA into malonyl-coenzyme A is the speed-limiting step in hepatic DNL, catalyzed by ACC (McGarry et al., 1977). ACC has two isoforms, ACC1 and ACC2, which work together to regulate the liver’s oxidation and synthesis of fatty acids. Moreover, the two ACC isoforms further increased lipid accumulation by increasing lipogenesis via DNL and by inhibiting lipid oxidation via carnitine palmitoyltransferase 1 (CPT1) (McGarry et al., 1977). HSCs necessitate glycolysis and DNL for activation. The exact mechanism by which DNL suppression prevents oxidative phosphorylation and glycolysis remains unknown. ACC inhibitors impede the capacity of HSCs to initiate glycolysis and oxidative phosphorylation in response to TGF-β, hence obstructing the metabolic transitions essential for HSC activation via both pathways. Furthermore, ACC inhibitors diminish lipid accumulation in hepatocytes and may mitigate fibrosis by reducing hepatocellular lipotoxicity and inhibiting HSCs activation (Bates et al., 2020). Despite the ambiguity surrounding their mechanism of action, ACC inhibitors demonstrate a beneficial impact on liver fibrosis. Notably, certain ACC inhibitors, such as Firsocostat in combination with the FXR agonist cilofexor, have advanced to clinical trials and have proven effective in improving key markers of non-alcoholic steatohepatitis (NASH) activity, including edema, inflammation, and steatosis (Loomba et al., 2021). Firsocostat also reduced the fibrosis-selective marker TIMP-1, thereby effectively ameliorating fibrosis (Loomba et al., 2018). Therefore, it has good prospects for clinical application. The latest meta-analysis shows that while ACC inhibitor monotherapy can improve steatosis and inflammation, it does not significantly improve the stage of liver fibrosis. In addition, the risk of hypertriglyceridemia is significantly elevated; this safety concern suggests that the clinical use of ACC inhibitors may require combination therapy or further optimization (Hasanatuludhhiyah et al., 2025). Furthermore, studies have shown that inhibiting ATP-citrate lyase (ACLY) reduces DNL in HSCs, thereby suppressing their activation and alleviating liver fibrosis. Furthermore, experiments involving exogenous fatty acid supplementation partially reversed the anti-activating effects of ACLY inhibitors, indicating that blocking endogenous lipid synthesis in HSCs is a key mechanism underlying the antifibrotic action of ACLY inhibitors (Morrow et al., 2022).

Stearoyl-CoA desaturase1 (SCD1) is another crucial enzyme in lipogenesis, responsible for converting lipotoxic saturated fatty acids into monounsaturated fatty acids (MUFAs). SCD1 was found to be overexpressed in activated HSCs and hepatocellular carcinoma cells among patients, as well as in rodent HSCs and murine liver TICs (Lai et al., 2017).The Wnt/β-catenin pathway enhances SCD1 transcription, while MUFAs generated by SCD1 stabilize β-catenin, creating a positive feedback loop that promotes liver fibrosis and tumor progression (Lai et al., 2017). Compared with non-tumor tissues, patients with hepatocellular carcinoma had significantly higher SCD1 levels. It demonstrated a positive correlation with tumor status and a negative correlation with patient survival time (Lai et al., 2017). Consequently, SCD1 inhibition diminished HSC activation and TIC self-renewal in mice, thereby mitigating liver fibrosis and carcinogenesis.

Moreover, it was demonstrated that in mice, arbutin (GA) reduced lipid accumulation induced by OA/PA and hepatic steatosis brought on by a high-fat diet (HFD). Hepatocytes activate the AMPK-ACC-PPARα pathway, inhibiting DNL, enhancing β-oxidation, improving mitochondrial function, and facilitating lipid metabolic reprogramming (Zhang et al., 2023).In the high-fat diet-induced mouse NAFLD model, AMPK activation significantly improved both NASH and fibrosis (Zhang et al., 2023). The role of the AMPK-ACC-PPARα axis in the reprogramming of lipid metabolism in HSCs and the potential protective impact of AMPK activation against fibrosis in mice models of hepatic fibrosis require further investigation.

#### 2.2.3 Accumulation of unbound cholesterol

The accumulation of free cholesterol stimulates HSC activity, thereby expediting the progression of liver fibrosis (Tomita et al., 2014b). Studies have shown that feeding mice a diet rich in fat and cholesterol leads to the accumulation of free cholesterol in HSCs and increased hepatic fibrosis (Gilgenkrantz et al., 2021). In the BDL and CCl_4_ models, free cholesterol concentrations in HSCs were markedly elevated in mice subjected to a high cholesterol diet compared to those on a control diet, and hepatic fibrosis, along with HSC activation, rose substantially in the cohort adhering to a high-cholesterol diet compared to the control group (Teratani et al., 2012). Acyl-CoA: cholesterol Acyltransferase 1(ACAT1) catalyzes the conversion of free cholesterol into cholesteryl esters; therefore, in a liver fibrosis mouse model, ACAT1 deficiency significantly exacerbated liver fibrosis but did not affect the extent of hepatic injury as well as hepatocyte inflammation (Tomita et al., 2014a). Deletion of ACAT1 markedly elevated Free cholesterol buildup inside HSCs, subsequently enhancing Toll-Like Receptor 4(TLR4) levels. Research demonstrated that Mice with a mutant form of TLR4 in C3H/HeJ do not experience exacerbated liver fibrosis when subjected to a high cholesterol diet, and HSCs with the TLR4 mutation similarly remain unactivated by the accumulation of free cholesterol (Teratani et al., 2012; Tomita et al., 2014a).

Furthermore, another study indicated that a single-nucleotide polymorphism (SNP) in the structural domain of the protein-like phospholipase containing the 3(PNPLA3) gene, resulting in the Ile148Met variant, was correlated with heightened histologic disease severity, fibrosis, and hepatocellular carcinoma (HCC) progression in individuals with NAFLD (Romeo et al., 2008; Valenti et al., 2010). Research demonstrated that the I148M PNPLA3 variant-carrying HSCs stimulate increased hepatic fibrogenesis. Furthermore, this PNPLA3 gene variant induces the phosphorylation of PPARγ at specific inhibitory residues, thereby downregulating its transcriptional activity and potentially disrupting downstream functions associated with both LXRs. Decreased LXR signaling diminishes intracellular cholesterol efflux and DNL, thereby exacerbating the buildup of free and total cholesterol (Bruschi et al., 2019). Targeted reduction of cholesterol accumulation in HSCs markedly diminishes hepatic fibrosis in a murine model (Berod et al., 2014).

In summary, reprogramming of lipid metabolism is an essential driver of HSC activation, transitioning from lipid droplet storage (predominantly retinol) to energy provision via fatty acid oxidation, leading to the aberrant upregulation of DNL and the buildup of free cholesterol, which exacerbates lipid accumulation. This reprogramming satisfies the substantial energy requirements for HSC activation and further facilitates the progression of liver fibrosis.

### 2.3 Glutamine metabolism and HSC activation

In addition to glycolysis and lipid metabolism, glutamine metabolism can also facilitate HSC activation. Glutamine metabolism has become a crucial source of ATP for HSCs’ phenotypic transition, enabling the shift to a myofibroblast phenotype (Du et al., 2018). Glutamine undergoes degradation in two primary stages. Initially, the enzyme glutaminase converts glutamine to glutamate. After that, glutamate is changed into α-ketoglutarate, which can fulfill the energy demands for the phenotypic transformation of HSCs by augmenting the activity of the tricarboxylic acid cycle (Du et al., 2018).

Activated HSCs and patient liver specimens with chronic liver damage, NASH, and severe liver fibrosis exhibit elevated glutaminase expression (Li et al., 2017; Du et al., 2018, Du et al., 2020). When initial quiescent HSCs undergo transdifferentiation into myofibroblasts, the expression of genes involved in glutamine metabolism is elevated, and the suppression of glutamine metabolism impedes transdifferentiation. Inhibiting glutamine metabolism in myofibroblasts significantly suppresses mitochondrial respiration, cellular proliferation, migration, and fiber production. The enzyme glutaminase is the one that limits the rate at which glutamine is hydrolyzed. In a liver fibrosis model in mice, glutaminase inhibition successfully prevented myofibroblast formation and fibrosis progression. Silencing the hedgehog signaling intermediary Smoothened(SMO) or Yes-Associated Protein 1(YAP) suppressed glutaminase expression; hence, Hedgehog and YAP inhibitors impeded glutamine metabolism and further reduced HSC activation. A study by Du et al. systematically evaluated the role of CB-839 in HSCs (Du et al., 2018).In HSCs, treatment with CB-839 inhibits glutaminase activity, reduces glutamine degradation, and consequently decreases mitochondrial respiration, cell proliferation, and migration capacity; In a mouse model of liver fibrosis induced by CCl_4_ and BDL, treatment with CB-839 significantly reduced hepatic collagen deposition and α-SMA expression; In addition, the study found that glutaminase (GLS1) expression was significantly elevated in activated human HSCs and was positively correlated with the stage of fibrosis in the livers of patients with NASH (Du et al., 2018). However, another study showed that while CB-839 alone had only a limited inhibitory effect on the proliferation of human HSCs, its anti-fibrotic effect was enhanced when used in combination with 2-DG (Smith-Cortinez et al., 2020). Currently, CB-839 is primarily in the clinical trial phase for cancer; its clinical translation for liver fibrosis requires further research (Song et al., 2018).

## 3 Matrix stiffness regulates cellular metabolism

In recent years, the mechanics of machinery have garnered heightened interest, with recent suggestions indicating that mechanical signals might modulate cellular metabolism, which in turn could further influence the development of liver fibrosis. The relationship between machinery mechanics and cellular metabolism remains underexplored; thus, we will examine the interactions between these domains, with an emphasis on the mechanical characteristics of the microenvironment. Our objective is to uncover new connections among machinery mechanics, signaling, and cellular metabolism, thereby generating innovative concepts and strategies to address liver fibrosis. HSCs remain quiescent without abnormal ECM deposition in the physiological state. Conversely, hepatic matrix stiffness increases with the progression of liver fibrosis, which is correlated with substantial ECM deposition, aberrant collagen alignment and organization, cellular stiffening, heightened matrix cross-linking, and inadequate ECM degradation due to an imbalance between matrix metalloproteinases and their tissue inhibitors (Chen et al., 2019). Alterations in ECM stiffness induce modifications in the mechanical microenvironment, enabling mechanotransduction to convert this information into biological signals that prompt cellular responses.

As liver fibrosis progresses, the stiffness of the liver’s extracellular matrix gradually increases. Ryoo et al (Ryoo et al., 2024) conducted a study using PEG microgel scaffolds and found that a 6 kPa microgel can simulate the matrix stiffness of early- to mid-stage liver fibrosis. A microgel at 25 kPa indicates advanced fibrosis. These stiffness ranges are highly correlated with physiological states. Furthermore, it has been demonstrated to differentially regulate the activation status and metabolic activity of HSCs.

In the early stage of liver fibrosis, increased matrix stiffness is primarily a consequence of HSC activation and excessive ECM production, during which metabolic reprogramming of HSCs themselves plays a pivotal driving role. However, in the late stage of disease progression, the persistently stiff ECM acts as a mechanical signal that feeds back to further promote HSC metabolic reprogramming (Lachowski et al., 2019; Zhao et al., 2025), thereby amplifying HSC activation and liver fibrosis to form a pathological positive feedback loop (Olsen et al., 2011). Of particular note, matrix stiffness in the late phase may also alter global hepatic metabolism, thereby creating a pro-fibrotic microenvironment that indirectly aggravates HSC activation (Zou et al., 2025). This indirect effect represents an emerging area of research and a key direction for future investigations.

### 3.1 ECM mechanotransduction regulates glucose metabolism.

Research indicates that the concentration of the pivotal rate-limiting enzyme in glycolysis, PFK, can be modulated by mechanical forces. Within bronchial epithelial cells, stress fibers sequester the ubiquitin ligase tripartite motif-containing protein 21 (TRIM21) on a stiff ECM, thereby preventing its ability to promote the destruction of PFK40 (Park et al., 2020). Conversely, on a softer ECM, stress fibers are suppressed, enabling TRIM21 to target PFK and induce its breakdown, therefore diminishing the glycolytic rate. Conversely, the aforementioned reduction in glycolytic rate did not elicit a compensatory augmentation in mitochondrial biogenesis, indicating that the two energy production modalities were not previously significantly correlated; specifically, the cell’s capacity to metabolize the total energy generated is limited when situated on a soft ECM (Romani et al., 2021). A soft ECM can diminish glycolysis rates by facilitating the breakdown of PFK, thereby lowering glycolytic activity.

Another study showed (Hu et al., 2016) that within fibroblasts, another key enzyme in glycolysis, aldolase, which is sequestered upon binding to f-actin (Pirovich et al., 2021)and released upon actin remodeling mediated by Ras-associated C3 botulinum toxin substrate 1(RAC1), causes increased glycolysis. It has been demonstrated (Klein et al., 2009) that the remodeling activity of actin mediated by RAC1 is markedly diminished on a soft ECM, resulting in a decrease in glycolysis due to the inhibition of aldolase release.

In contrast to the PFK and aldolase mechanisms described above, Fujisawa et al (Fujisawa et al., 2020)observed an increase in compensatory glycolysis in HSC spheroids. They found that, despite elevated levels of glycolytic intermediates, the levels of TCA cycle intermediates (α-ketoglutarate and succinate) were significantly reduced. This indicates that mitochondrial oxidative phosphorylation is inhibited. This contrasts with the mechanism observed by Park et al. in fibroblasts, in which “a stiff matrix directly enhances glycolysis via TRIM21/PFK.” On a stiff ECM, enhanced glycolysis is an active process driven by mechanical stretching; On softer ECM, enhanced glycolysis serves as a compensatory response to mitochondrial inhibition.

**Figure 2:**
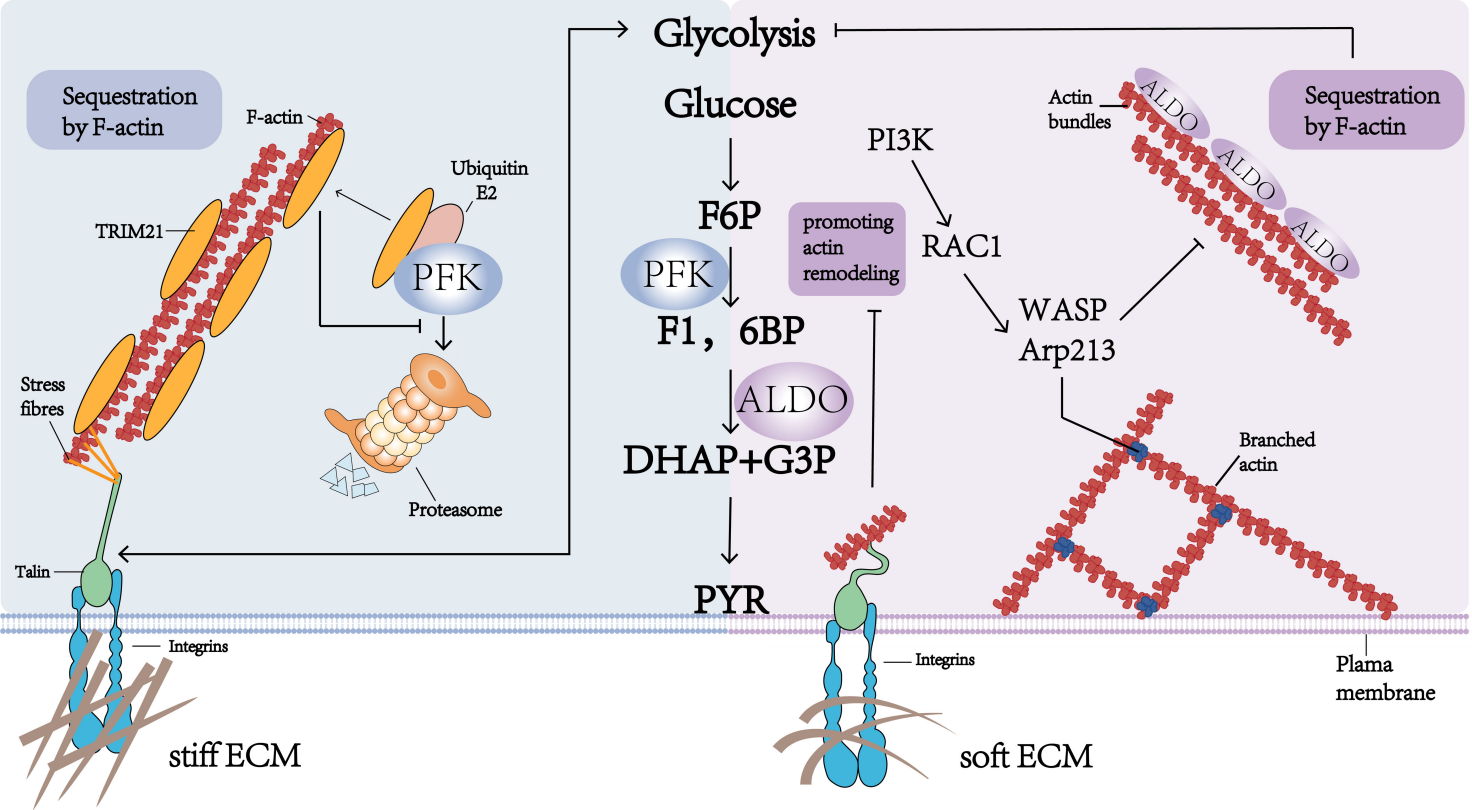
ECM mechanotransduction regulates glucose metabolism. Left: A stiff ECM drives glycolysis to occur, and the above process happens via ubiquitin ligase TRIM21-mediated regulation of PFK proteolytic cleavage. F-actin sequesters TRIM21 when the cell is on a stiff matrix, thereby inhibiting PFK degradation. Right: Through the sequestration of the glycolytic enzyme aldolase (ALDO), which may be released in response to RAC1-mediated skeleton remodeling, the actin cytoskeleton can control gluconeogenesis, thereby enhancing glycolysis, and the above process may be associated with the Wiskott-Aldrich Syndrome Protein (WASP) and the actin-associated protein 2/3 (Arp2/3) complexes.

The TRIM21-PFK and RAC1-aldolase pathways, established in other cell types, await direct validation in HSCs. Moreover, the compensatory glycolysis observed in HSC spheroids suggests unique metabolic adaptations to mechanical cues (**Figure 2**). Future studies should prioritize direct mechanistic validation in HSCs, including whether PFK degradation or aldolase release occurs in response to ECM stiffness, and how these pathways integrate with mitochondrial function to regulate HSC activation and fibrosis.

### 3.2 ECM mechanotransduction regulates lipid synthesis

**Figure 3:**
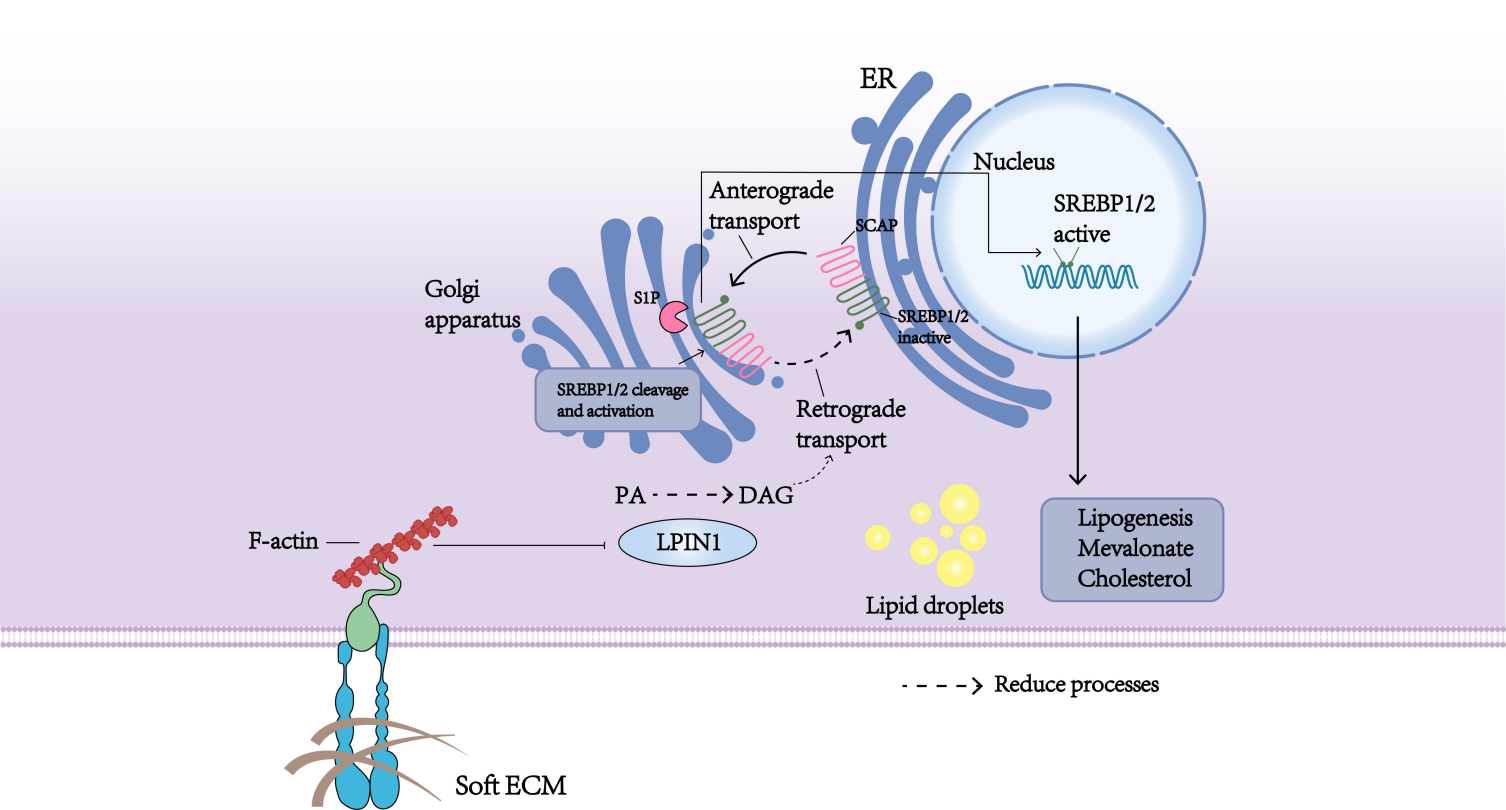
ECM mechanotransduction regulates lipid synthesis. When cells are on the soft ECM, their reduced cytoskeletal tension inhibits the activity of PA and LPIN1. It reduces the content of DAG, which induces an imbalance in the cis-retrograde translocation of SREBP1/2 and its transport regulator, SREBP cleavage-activating protein (SCAP), between the Golgi and the ER, leading to a massive Protease S1P cleaves SREBP1 and SREBP2 once they accumulate in the Golgi.

It has been shown that within stem cells, ECM stiffness regulates lipid synthesis (Bertolio et al., 2019; Romani et al., 2019). Decreased ECM stiffness leads to diminished actin contractility, so altering the Golgi apparatus’s surrounding actin’s stiffness. It accomplishes this by suppressing phosphatidylinositol 1 (LPIN1) activity, producing a substantial reduction in phosphatidic acid (PA) synthesis and Diacylglycerol(DAG) levels. The accumulation of PA and the decrease in DAG content result in a notable decline in the recruitment of ADP-Ribosylation Factor 1(ARF1). This transporter governs reverse transport from the Golgi to the endoplasmic reticulum(ER), Thus, the balance between paracrine and retrograde transport of the SREBP-SCAP complexes is interrupted (Singh et al., 2018; Romani et al., 2019), resulting in a substantial decrease in sterol regulatory element-binding protein1/2 (SREBP1/2) in the Golgi apparatus, subsequently cleaved by the protease S1P, which modifies the gene expression of lipid synthesis by releasing transcriptionally active cytoplasmic SREBP fragments into the cellular nucleus. Research indicates that the SREBP-mediated lipid synthesis genes mentioned above promote the accumulation of diacylglycerol, triglycerides, and cholesterol at the expense of membrane phospholipid production, which is characteristic of rapid cell proliferation, resulting in a substantial accumulation of free lipid droplets (Bertolio et al., 2019; Romani et al., 2019) (**Figure 3**). Research indicates that levels of triglycerides and cholesteryl esters are markedly diminished in human scars, suggesting that physiological or pathological alterations that lead to tissue rigidity may influence SREBP activity and lipid metabolism. In human pluripotent stem cells, soft ECM can promote lipid synthesis and accumulation by activating SREBP (Romani et al., 2019).

It is worth noting that Fujisawa et al (Fujisawa et al., 2020)found in their research on HSC spheres that the expression of SREBP1 was upregulated in the spheres, and the levels of saturated fatty acids and membrane phospholipids increased, indicating that HSCs in the softer ECM also initiated *de novo* lipid synthesis. This is consistent with the findings of Romani et al. in fibroblasts. However, in Fujisawa’s study, HSC spheres showed inhibited proliferation and low expression of α-SMA. It is suggested that lipid synthesis in softer ECM is mainly used to maintain membrane structure rather than support proliferation.

The correlation of the lipid metabolic response with HSCs and other cell types remains ambiguous; nonetheless, the studies mentioned above indicate that the presence of a soft ECM, characterized by reduced proliferation and heightened lipogenesis, augments the body’s food reserves to bolster disease resistance. The aforementioned investigate a general correlation between cellular lipid metabolism and ECM mechanosignaling; however, in HSCs, the relationship between ECM stiffness and lipid metabolism, along with its association with HSC activation, requires more exploration.

In summary, ECM stiffness facilitates cellular metabolic reprogramming via integrin-cytoskeletal mechanosignaling. Elevated stiffness enhances glycolysis to satisfy the energy requirements of rapid proliferation, stimulates lipid metabolism for the synthesis of membrane phospholipids indicative of swift cell division, and supplies structural precursors for membranes. While the relationship between matrix stiffness-mediated reprogramming of cellular metabolism and HSCs remains ambiguous, activated HSCs in the liver are the principal producers of ECM and are pivotal in the augmentation of matrix stiffness. According to traditional viewpoints, elevated matrix stiffness is not only a harmful consequence of liver fibrosis but is also recognized as a critical element in the pathological progression of liver fibrosis (Chen et al., 2019). It has been shown that a stiffer matrix induces differentiation of portal fibroblasts (Li et al., 2007) and HSC (Olsen et al., 2011) to myofibroblasts. Therefore, a pathological positive feedback loop is formed: HSC activation-ECM deposition and increased stiffness-enhanced mechanotransduction-further HSC metabolic reprogramming and activation-more severe fibrosis. These effects can be categorized as direct and indirect. Directly, elevated stiffness enhances glycolysis to satisfy the energy requirements of rapid proliferation, stimulates lipid metabolism for the synthesis of membrane phospholipids. Indirectly, alterations in global hepatic metabolism induced by a stiff ECM create a pro-fibrotic microenvironment that further aggravates HSC activation. We can conduct subsequent studies on the vicious cycle in which increased matrix stiffness promotes HSC metabolic reprogramming and activation, leading to excessive ECM deposition, which in turn further hardens the matrix.

## 4 Summary and outlook

Further research on HSC activation and their intracellular metabolism has revealed an effective approach for treating liver fibrosis and developing new medications: targeting HSC metabolism. Identifying additional treatment targets for liver fibrosis requires an understanding of the material and energetic alterations during HSC activation, as well as the associated metabolic pathways, substrates, and critical enzymes.

Mechanosignaling in cell biology constitutes a complex network of physical stimuli, including ECM stiffness, tensile or compressive forces, fluid shear, and intercellular forces. To date, the majority of *in vitro* research has focused on mechanotransduction associated with cell-ECM and cell-cell interactions. This article similarly examines the correlation between ECM stiffness and cellular metabolic reprogramming, while the association between other forms of mechanomechanics and cellular metabolism remains to be explored further. The existence of the aforementioned machinery mechanisms within HSCs, facilitating cellular metabolic reprogramming and subsequently promoting fibrogenesis, necessitates further investigation.

As a review article, the mechanisms of metabolic reprogramming discussed here are primarily based on preclinical research data, and some key conclusions lack direct experimental evidence; In addition, this paper discusses the roles of glycolysis, lipid metabolism, and glutamine metabolism in HSC activation, but does not provide a systematic analysis of the cross-regulation and compensatory mechanisms among these pathways; Regarding the theory that ECM stiffness regulates HSC metabolism through mechanical transmission, most of the current evidence remains at the level of correlation; the causal relationship and the consistency between in vitro models and in vivo pathological conditions still need to be verified; Finally, many of the metabolic inhibitors mentioned in this article are still in the preclinical or early clinical trial stages, and their efficacy and safety in patients with liver fibrosis require further support from human data.

Future research may favor multi-targeted therapy for liver fibrosis as a more advantageous approach. First, given that HSC activation depends on glycolysis, lipid metabolism, and glutamine metabolism, the efficacy of single-target inhibition may be limited due to compensatory upregulation of metabolic pathways, Therefore, it is necessary to systematically evaluate multi-target combination therapy strategies in in vivo fibrosis models, such as the combination of glycolysis inhibitors with glutaminase inhibitors, or further combination with matrix-modifying agents (such as LOXL2 inhibitors) to break the vicious cycle in which matrix stiffness drives HSC activation and the progression of fibrosis (**Table 1**). Second, given the risk of systemic side effects associated with existing metabolic inhibitors, it is crucial to develop HSC-specific targeted delivery strategies. By leveraging the physiological and pathological characteristics of HSCs, various ligand-receptor-mediated targeting systems have already been established: 1. Vitamin A and its active form, retinoic acid, can specifically recognize and activate HSCs through the retinol-binding protein receptor pathway and are currently the most widely used HSC-targeting ligands (Baglieri et al., 2019; Liu et al., 2022); 2. Integrin αvβ3 is highly expressed in activated HSCs, and the RGD sequence to which it specifically binds can be engineered as a targeting ligand (Beljaars et al., 2000; Zhou et al., 2004); 3. The hyaluronan receptor CD44 is highly expressed on the surface of activated HSCs, and hyaluronan itself can serve as a targeting ligand to mediate nanodelivery (Fang et al., 2011; Yoshizaki et al., 2023); 4. The expression of PDGFR-β in HSCs is significantly higher than in other cell types. The cyclic peptide pPB cyclic peptide(pPB), designed based on the receptor-binding domain of the PDGFR-β chain, can specifically recognize PDGFR-β, enabling targeted delivery to HSCs (Beljaars et al., 2003; Gao et al., 2020). The aforementioned nanodelivery systems, antibody-drug conjugates, and prodrug strategies responsive to the fibrotic microenvironment hold promise for achieving cell-type-specific metabolic interventions. They also offer new avenues for the precision treatment of liver fibrosis.

**Table 1:** Key studies on metabolic reprogramming in hepatic stellate cell activation.

Metabolic pathway	Key regulator	Experimental model	Main finding
Glycolysis	PFKFB3	Human/Mouse HSCs, BDL mouse models	PFKFB3 interacts with CPEB4 to enhance glycolysis and HSC activation; PFKFB3 inhibition (3PO) reduces fibrosis (Mejias et al., 2020).
Glycolysis	PKM2	Human HSCs, CCl_4_ mouse model	PKM2 tetramerization inhibits HSC activation; PKM2 agonist TEPP-46 attenuates liver fibrosis (Zheng et al., 2020).
Glycolysis	GLUT1	Human HSCs, fibroblast cell lines	TGF-β induces GLUT1 expression; GLUT1 inhibition diminishes TGF-β-mediated fibrogenesis (Andrianifahanana et al., 2016).
Lipid metabolism	Autophagy	Mouse HSCs, CCl_4_/TAA models	Autophagy releases fatty acids from lipid droplets to fuel β-oxidation and HSC activation (Hernández-Gea et al., 2012).
Lipid metabolism	ACC	Primary HSCs	ACC inhibition disrupts metabolic reprogramming required for HSC activation (Bates et al., 2020).
Lipid metabolism	ACLY	Human HSCs	ACLY inhibition reduces de novo lipogenesis in HSCs, suppressing activation and alleviating fibrosis (Morrow et al., 2022).
Lipid metabolism	SCD1	Human/Mouse HSCs	SCD1-driven MUFA synthesis promotes Wnt/β-catenin signaling, creating a positive feedback loop (Lai et al., 2017).
Cholesterol metabolism	ACAT1	Mouse HSCs, high-cholesterol diet	Free cholesterol accumulation via ACAT1 deficiency enhances TLR4 expression and aggravates fibrosis (Teratani et al., 2012; Tomita et al., 2014a).
Cholesterol metabolism	PNPLA3	Human HSCs, NAFLD patients	I148M PNPLA3 variant impairs LXR signaling, exacerbating free cholesterol accumulation and fibrosis (Bruschi et al., 2019).
Glutamine metabolism	GLS1	Human HSCs, CCl_4_/BDL mouse models	Hedgehog-YAP signaling regulates glutaminolysis; GLS1 inhibitor CB-839 suppresses HSC activation and fibrosis (Du et al., 2018).
Glutamine metabolism	GLS1 + 2-DG	Human HSCs	CB-839 alone has a limited effect on human HSC proliferation; combination with 2-DG enhances anti-fibrotic efficacy (Smith-Cortinez et al., 2020).
Mechanotransduction & Metabolism	TRIM21/PFK	Bronchial epithelial cells	Stiff ECM sequesters TRIM21 to prevent PFK degradation, enhancing glycolysis (requires validation in HSCs) (Park et al., 2020).
Mechanotransduction & Metabolism	Aldolase/RAC1	Fibroblasts	RAC1-mediated actin remodeling releases aldolase from F-actin to enhance glycolysis (requires validation in HSCs) (Klein et al., 2009).
Mechanotransduction & Metabolism	Lipin-1/SREBP	stem cells; Human HSC spheroids	Soft ECM suppresses Lipin-1 activity, altering SREBP trafficking and promoting lipid accumulation (Bertolio et al., 2019; Romani et al., 2019); Soft ECM promotes SREBP1 upregulation and de novo lipid synthesis (Fujisawa et al., 2020).

In summary, this review makes the following innovative contributions: First, it challenges the traditional view that matrix stiffness is merely a passive consequence of liver fibrosis and, for the first time, proposes a new concept in which it acts as an active driving factor, forming a pathological positive feedback loop with HSC metabolic reprogramming; Second, the study integrated the roles of three metabolic pathways: glycolysis, lipid metabolism, and glutamine metabolismin HSC activation, revealing that these pathways regulate each other synergistically rather than in isolation; Third, linking mechanical transduction with metabolic reprogramming provides a unified theoretical framework for understanding how mechanical signals regulate HSC metabolism. Building on this, we propose multi-target combination therapy strategies and research directions for the development of HSC-specific targeted delivery systems.

## Glossary

HSC: Hepatic stellate cells
ECM: extracellular matrix
FDA: Food and Drug Administration
α-SMA: α-smooth muscle actin
TIMPs: tissue inhibitors of metalloproteinases
MMP: matrix metalloproteinase
TCA: tricarboxylic acid cycle
GLUT1: glucose transporter1
PKM2: Pyruvate Kinase M2
CCND1: Cyclin D1
PFKFB3: Fructose-2, 6-Bisphosphatase 3
MYC: MYC Proto-Oncogene
CPEB4: Cytoplasmic Polyadenylation Element Binding Protein 4
TGF-β: transforming growth factor-β
ACAT1: acetyl coenzyme A acetyltransferase
FXR: farnesol receptor
LXR: liver X receptor
TLR4: toll-like receptor 4
HK2: hexokinase2
PFK1: phosphofructokinase-1
GLUTs: glucose transporters
GAPDH: glyceraldehyde-3-phosphate dehydrogenase
MCD: methionine-choline-deficient
BDL: bile duct ligation
F2: 6BP, fructose-2, 6-bisphosphate
PDGFR-β: Platelet-Derived Growth Factor Receptor Beta
VEGF: Vascular Endothelial Growth Factor
COL1A1: collagen type I alpha 1 chain
PDGF: Platelet-Derived Growth Factor
RAB31: Ras-Related Protein Rab-31
EVs: extracellular vesicles
HIF-1α: Hypoxia-inducible factor-1α
IL-1β: Interleukin-1 Beta
TNF-α: Tumor necrosis factor-α
HRE: Hypoxia Response Element
LDH: lactate dehydrogenase
PDK1: pyruvate dehydrogenase kinase1
LD: lipid droplets
REs: Retinyl esters
ADRP: adipocyte differentiation-related protein
ATGL: adipose triglyceride lipase
CGI-58: comparative gene identification-58
L-FABP: liver-fatty acid-binding protein
RARβ: Retinoic Acid Receptor Beta
RXRα: Retinoid X Receptor Alpha
GFAP: Glial Fibrillary Acidic Protein
PPAR-γ: Peroxisome Proliferator-Activated Receptor Gamma
HSP-47: Heat Shock Protein 47
DNL: *de novo* lipogenesis
MASLD: Metabolic dysfunction-associated steatotic liver disease
ACC: Acetyl-CoA carboxylase
acetyl-CoA: acetyl-coenzyme A
CPT1: carnitine palmitoyltransferase 1
NASH: non-alcoholic steatohepatitis
ACLY: ATP-citrate lyase
SCD1: Stearoyl-CoA desaturase1
MUFAs: monounsaturated fatty acids
GA: arbutin
HFD: high-fat diet
ACAT1: Acyl-CoA, cholesterol Acyltransferase 1
TLR4: Toll-Like Receptor 4
SNP: single-nucleotide polymorphism
PNPLA3: protein-like phospholipase containing the 3
HCC: hepatocellular carcinoma
SMO: Smoothened
YAP: Yes-Associated Protein 1
GLS1: glutaminase
TRIM21: tripartite motif-containing protein 21
RAC1: Ras-associated C3 botulinum toxin substrate 1
ALDO: aldolase
WASP: Wiskott-Aldrich Syndrome Protein
Arp2/3: actin-associated protein 2/3
LPIN1: phosphatidylinositol 1
SREBP1/2: sterol regulatory element-binding protein1/2
SCAP: srebp cleavage-activating protein
DAG: Diacylglycerol
ARF1: ADP-Ribosylation Factor 1
ER: endoplasmic reticulum
pPB: pPB cyclic peptide
